# Current and Emerging Therapies for Metastatic Castration-Resistant Prostate Cancer (mCRPC)

**DOI:** 10.3390/biomedicines9091247

**Published:** 2021-09-17

**Authors:** Iván Henríquez, Mack Roach, Todd M. Morgan, Alberto Bossi, Junior A. Gómez, Oscar Abuchaibe, Felipe Couñago

**Affiliations:** 1 Department of Radiation Oncology, Hospital Universitario Sant Joan, 43204 Reus, Spain; junioranderson.gomez@gmail.com; 2 UCSF Helen Diller Family Comprehensive Cancer Center, Department of Radiation Oncology, San Francisco, CA 94143, USA; mack.roach@ucsf.edu; 3 Rogel Cancer Center, Department of Urology, University of Michigan, Ann Arbor, MI 48109, USA; tomorgan@med.umich.edu; 4 Prostate Brachytherapy Unit, Department of Radiation Oncology, Genito Urinary Oncology, Goustave Roussy, 94805 Paris, France; Alberto.BOSSI@gustaveroussy.fr; 5 Virgilio Galvis Ramirez Cancer Centre, Department of Radiation Oncology, Bucaramanga 681004, Colombia; oaabucha@hotmail.com; 6 Department of Radiation Oncology, Clinical Department, Faculty of Biomedicine, Hospital Universitario Quirónsalud Madrid, Hospital La Luz, Universidad Europea, 28223 Madrid, Spain; fcounago@gmail.com

**Keywords:** mCRPC, prostate cancer, androgen deprivation therapy, novel therapies

## Abstract

Metastatic castration-resistant prostate cancer (mCRPC) encompasses a heterogeneous wide range of molecular tumor behavior and a high risk of progression. Early detection and treatment are therefore crucial in these patients. Treatment has improved drastically in recent years and many novel therapeutic agents are currently under investigation. However, due to the rapidly changing therapeutic landscape in mCRPC, it is difficult for clinicians to keep up to date with the latest innovations in this area. In the present narrative review, we discuss the current and emerging therapies for mCRPC as well as the clinical and molecular factors that can help predict which patients are most likely to benefit from these novel agents.

## 1. Introduction

Prostate cancer (PCa) is one of the most common cancers in men. In the year 2020, an estimated 191,000 new cases of PCa were diagnosed in the United States (USA) with more than 33,000 deaths. In that same year, 449,761 new diagnoses were recorded in Europe, with more than 100,000 deaths [1,2].

At diagnosis, most patients have localized disease. However, 5% of patients are diagnosed with metastatic disease and 30−40% of patients will develop biochemical recurrence after treatment (surgery and/or radiotherapy) [2]. A substantial proportion of these patients will develop metastatic castration-resistant prostate cancer (mCRPC). The long-term prognosis for patients with mCRPC is poor, with a relatively short overall survival (OS), although survival varies highly depending on individual disease characteristics [2,3,4].

Since the introduction of docetaxel in 2004, there has been a surge in novel treatments for mCRPC, including new cytostatic agents, second-generation antiandrogens, bone-targeted therapies, immunotherapy, poly (adenosine diphosphate-ribose) polymerase (PARP) inhibitors, Akt inhibitors, and radioisotopes. These advances have improved survival in patients with mCRPC while also improving quality of life (QoL). 

Due to the rapidly changing therapeutic landscape in mCRPC, it is difficult for clinicians to keep up to date with the latest innovations in this area. Consequently, the aim of the present narrative review is to review the currently available evidence on novel and emerging therapies for the treatment of mCRPC. In recent years, new treatments have been developed for non-metastatic CRPC, but this type of CRPC is beyond the scope of the present narrative review.

## 2. Methods

### 2.1. Castration-Resistant Prostate Cancer

Most men with advanced disease eventually stop responding to traditional androgen-deprivation therapy (ADT) and are thus categorized as CRPC, which is defined as prostate cancer that progresses clinically, radiographically, or biochemically despite castration levels of serum testosterone. Imaging tests may be indicated to monitor for signs of distant metastases. Factors that determine how often imaging should be performed include individual risk, age, overall patient health, prostate-specific antigen (PSA) velocity, and Gleason score [5]. CRPC is defined as a documented rise in PSA ≥ 2 ng/mL, PSA values > 25% above nadir, PSA elevation in three consecutive determinations at least one week apart, and/or radiological progression in castrated patients with serum testosterone levels < 50 ng/dL (<1.7 nmol/L) [5,6].

### 2.2. Search Strategy, Data Acquisition, and Risk of Bias

We conducted a systematic review of the literature using the Embase, PubMed, and Scopus electronic databases. We searched for relevant articles published in those databases between January 2010 and December 2020 using the following search terms: “advanced prostate cancer”; “metastatic castration resistant prostate cancer”; “randomized studies”; “chemotherapy”; “androgen deprivation therapy”; “immunotherapy”; “second-generation antiandrogens”; “radiopharmaceuticals”; “PARP inhibitors”; “radioisotopes”; “AKT inhibitors”; and “bone-targeted therapy”. We included original articles published in English, studies reporting oncologic outcomes, biochemical failure, progression-free survival (PFS), cancer-specific mortality, OS, and/or patient-reported side effects. We included both randomized controlled trials (RCTs) and controlled (nonrandomized) clinical trials. We excluded cross-sectional studies, case series, case reports, and articles published by the same group of researchers with possible data overlap. We also checked the references included in the selected articles to locate other studies not identified in the initial search. The studies with the highest level of evidence were evaluated and selected for inclusion in this review by consensus agreement among the researchers. The Cochrane risk tool was used to evaluate the risk of bias in the clinical trials [7].

## 3. Current Treatments in mCRPC

Most men with PCa will eventually develop disease progression despite castration. Two trials have shown only a marginal survival benefit for patients remaining on LHRH analogues during second- and third-line therapies [8,9]. However, in the absence of prospective data, the modest potential benefits of continuing agonists or antagonists to suppress testosterone to castration levels outweigh the minimal risks of treatment. Since then, all clinical trials conducted at CRPC include ADT as part of baseline treatment (Table 1).

### 3.1. Chemotherapy

#### 3.1.1. Docetaxel

Since the year 2004, several clinical trials have been conducted to evaluate the efficacy of docetaxel, a cytotoxic agent of the taxane family, in mCRPC. In the first phase III trial, TAX327 [10], a total of 1006 patients with mCRPC were randomized to receive docetaxel + prednisone (DP) in two different regimens (three times weekly at 75 mg/m^2^, or weekly at 30 mg/m^2^) or mitoxantrone + prednisone (MP). OS was longer in the three-weekly DP arm versus the MP arm (median survival: 18.9 vs. 16.5 months, respectively; hazard ratio [HR]: 0.76, *p* = 0.009). However, no differences in OS were observed between the weekly DP arm and MP. Secondary outcomes such as prostate-specific antigen (PSA) response rate, pain reduction, and QoL measured by the FACT-P questionnaire, were also better in the three-weekly DP arm. The most common treatment-related toxicities associated with DP were alopecia (65%), asthenia (53%), nausea (42%), diarrhea (32%), and neuropathy (30%). The incidence of grade 3/4 neutropenia was 32%, with 26% of patients experiencing at least one severe adverse event.

Another phase III trial in the same subgroup of patients compared docetaxel + estramustine (DE) versus MP [11], showing that OS was better in the group that received DE. However, due to the severe toxicity profile of estramustine, which includes a risk of thrombotic events and cardiovascular toxicity, this agent is no longer used. In an attempt to find a regimen with more manageable toxicity, a phase II trial was performed to compare the classical three-weekly regimen of 75 mg/m2 of DE compared to a dose of 50 mg/m^2^ delivered on days one and 14 of the 28-day cycle [12]. All patients received 10 mg of oral prednisolone daily. The primary outcome measure—time to treatment failure—was superior in the twice-monthly DP arm (5.6 vs. 4.9 months; HR 1.3, 95% confidence interval [CI]: 1.1–1.6, *p* = 0.014), with a longer OS (19.5 vs. 17 months; HR 1.4, 95% CI: 1.1–1.8, *p* = 0.021). There was also less toxicity in the twice-monthly arm, with a decrease in both hematologic and digestive toxicity (diarrhea, nausea). Therefore, the five-weekly DP regimen can be considered a valid treatment option, especially in frail patients given the important need to limit the toxicity in these patients.

#### 3.1.2. Cabazitaxel

Cabazitaxel is another member of the taxane family that has proven effective even in docetaxel-resistant cancers. The results of the pivotal phase III TROPIC trial were reported in 2010 [13]. In that trial, which included 775 patients with mCRPC who had received prior treatment with docetaxel, patients were randomized to cabazitaxel + prednisone (CP) or MP. OS was significantly longer in the CP arm (15.1 vs. 12.7 months, HR 0.7, *p* < 0.0001), with a greater PSA response and longer PFS. Severe toxicity associated with the CP regimen was mainly hematologic, most commonly neutropenia (94% of patients), grade 3/4 neutropenia (82%), and febrile neutropenia (8%). The most important non-hematologic toxicities were diarrhea (47% of patients; 6% grade 3–4), nausea (34%; 2% grade 3–4), and fatigue (37%; 5% grade 3–4). Based on those findings, CP became the standard treatment option for patients with progression after prior DP.

The phase III FIRSTANA trial was performed to evaluate whether cabazitaxel 20 mg/m^2^ (C20) or 25 mg/m^2^ (C25) was superior to docetaxel (75 mg/m^2^) in terms of OS as a first-line treatment of mCRPC. However, there were no significant differences between the two treatment regimens [14]. Median OS for the higher dose regimen (C25) was 25.2 months versus 24.3 months for docetaxel (HR 0.97; 95% CI: 0.82–1.16, *p* = 0.757). Another phase III trial was performed to assess the possibility of administering cabazitaxel at a lower dose (20 mg/m^2^ versus the standard dose of 25 mg/m^2^), as part of the PROSELICA study [15]. OS rates were similar, but with less toxicity in the low dose arm, thus indicating that the 20 mg/m^2^ dose was a safe and effective option in these patients.

### 3.2. Second-Generation Antiandrogens

The development of second-generation antiandrogens showed that a large percentage of prostate cancers remain dependent on androgen signaling, even at the low serum testosterone levels present after castration. As a result, the term “castration-resistant” came to replace the classical terms “hormone-refractory” or “hormone-independent”.

#### 3.2.1. Abiraterone

Abiraterone is an androgen biosynthesis inhibitor that functions by inhibiting two of the main enzymes (17-alpha-hydroxylase and 17,20-lyase) involved in PCa. These enzymes are necessary for androgen biosynthesis in testicular, suprarenal, and prostate tumor tissues. Given that abiraterone can lead to a rebound effect due to hyperactivation of the mineralocorticoid axis, it should be administered in combination with prednisone.

Continuous CYP17 inhibition raises levels of adrenocorticotropic hormone (ACTH), which increases steroid levels upstream of CYP17, including corticosterone and deoxycorticosterone. These raised upstream steroids prevent adrenocortical insufficiency but can result in a syndrome of secondary mineralocorticoid excess characterized by fluid retention, hypertension, and hypokalemia. This can be ameliorated by low-dose glucocorticoids, which decrease ACTH and steroids upstream of the CYP17 blockade. Prednisone is a synthetic corticosteroid metabolized to prednisolone (active form) in the liver [16].

Publication of the phase III trial COU-AA-301 in 2011 led to the approval of abiraterone as a treatment for mCRPC [16]. In that trial, 1195 patients who had progressed after treatment with docetaxel were randomized to abiraterone + prednisone or placebo + prednisone. Median OS (the primary outcome measure) was significantly longer in the abiraterone arm (14.8 vs. 10.9 months, HR 0.65, *p* < 0.001). In addition, secondary outcome measures (PSA response rate, time to PSA progression, and PFS) were all better in the abiraterone arm. Treatment-related toxicity, mainly due to hyperstimulation of the mineralocorticoid axis, included arterial hypertension (10%; grade 3–4: 1%), hypokalemia (17%: grade 3–4: 3%), and water retention (33%; grade 3–4: 2%). Patients in the abiraterone arm developed more heart conditions (mainly arrhythmias) than those who received placebo, but this difference was not statistically significant. Other adverse effects—including nausea, asthenia, pain, and elevated transaminase levels—were significantly more common in the treatment arm.

The results of another phase III trial (COU-AA-302) [17] were reported in 2012. That study included 1088 asymptomatic or mildly symptomatic chemotherapy-naïve (including docetaxel) patients diagnosed with mCRPC. Patients were randomized to receive abiraterone + prednisone or placebo + prednisone. The results showed a clear benefit for abiraterone in terms of both rPFS (16.5 vs. 8.3 months, HR 0.53, *p* < 0.001) and OS (34.7 vs. 30.3 months; HR 0.81; *p* = 0.0033). No new toxicities or a higher rate of previously described toxicities were observed with longer exposure to the drug. Based on these findings, abiraterone + prednisone became the new standard of care for mCRPC in patients with and without prior chemotherapy.

#### 3.2.2. Enzalutamide

Enzalutamide is an androgen receptor antagonist with three different mechanisms of action: (1) it prevents the binding of testosterone and its metabolites to the androgen receptor; (2) it prevents translocation into the nucleus, and (3) it blocks binding to DNA, thus inhibiting its activity as a transcription factor. Importantly, enzalutamide does not have any agonistic activity and induces apoptosis in prostate cancer cell lines.

The development of enzalutamide paralleled that of abiraterone. The first phase III trial of this agent in the treatment of mCRPC (the AFFIRM trial), reported in 2012, was conducted in a post-docetaxel setting in a large sample (n = 1199) of patients [18]. Patients were randomized to receive enzalutamide or placebo, with significantly longer OS in the treatment arm (18.4 vs. 13.6 months, HR 0.63, *p* < 0.001). Secondary outcome measures—including PSA response rate, time to PSA progression, and radiographic PFS—were all better in the patients who received enzalutamide. In terms of adverse effects, enzalutamide was associated with neurotoxicity (convulsions), but in less than 1% of patients. There was also a higher incidence of arterial hypertension (6% vs. 3%), asthenia (34% vs. 29%), and hot flushes (20% vs. 10%) in the treatment arm.

The results of the phase III PREVAIL trial were reported in 2014 [19]. That trial was performed to evaluate enzalutamide administered before docetaxel versus placebo, in a study design that was similar to the COU-AA-302 trial with abiraterone. Compared to placebo, a higher proportion of patients in the enzalutamide arm achieved radiographic PFS at 12 months (65% vs. 14%, HR 0.19, *p* < 0.001) and OS was also significantly longer in the experimental arm (32.4 vs. 30.2 months, HR 0.7, *p* < 0.001). The most common adverse events in the enzalutamide arm were asthenia, hot flushes, and hypertension, but there was no clear increase in convulsions in the treatment arm versus placebo. Based on the finding of that clinical trial, enzalutamide became one of the standard hormonotherapy options in patients with mCRPC regardless of prior treatment with docetaxel.

### 3.3. Radiopharmaceuticals

#### Radium-223

Radiopharmaceuticals—primarily beta emitters, such as strontium or samarium—have long been prescribed, mainly for the palliative treatment of bone pain. Although these radiopharmaceuticals were effective for pain relief, they had no impact on survival. Radium-223 is a calcium mimetic alpha emitter that accumulates in the bone with a very low capacity to penetrate surrounding tissues, but greater cytotoxic capacity due to its higher linear energy transfer.

In 2013, the results of the pivotal phase III ALSYMPCA trial comparing Radium-223 to placebo in patients with mCRPC (n = 921) with symptomatic bone involvement but no evidence of visceral involvement was reported [20]. Patients had previously received docetaxel or were not eligible to receive it. The primary outcome measure was OS, which was significantly better in the Radium-223 group (14.9 vs. 11.3 months, HR 0.7, *p* < 0.001). Secondary outcome measures—time to first symptomatic bone event, time to alkaline phosphatase elevation, and QoL [21]—were all significantly better in the experimental arm. Interestingly, the toxicity profile was also better in the Radium-223 arm, with less overall toxicity than in the placebo arm, except for thrombopenia (12%; 6% grade 3–4), neutropenia (5%; 3% grade 3–4) and diarrhea (25%; 2% grade 3–4). Based on these findings, Radium-223 became a standard treatment option in patients with mCRPC with bone metastases (but not visceral metastases).

The phase III ERA-223 trial [22] evaluated Radium-223 in combination with abiraterone and prednisone versus placebo for the treatment of asymptomatic or mildly symptomatic chemotherapy-naive mCRPC. The combined treatment significantly increased the fracture risk (28.6% vs. 11.4%) and also reduced the mean survival time (30.7 vs. 33.3 m) compared to placebo. Based on an unplanned analysis of the ERA-223 trial, the Pharmacovigilance Risk Assessment Committee of the European Medicines Agency (EMA) concluded that the combination of Radium-223 and abiraterone was contraindicated, leading to an official recommendation against this combination in the European Union (EU) [22,23]. in the EU, Radium-223 is now indicated for use after at least two prior lines of systemic therapy for mCRPC (excluding LHRH analogues) or for patients who are ineligible for other systemic treatments [24]. Notably, the indication for Radium-223 remains unchanged in many countries, including the United States, Canada, Switzerland, and Japan.

## 4. Emerging Treatments in mCRPC

Numerous different treatments have been evaluated for mCRPC, but none have demonstrated any overall survival benefit. Several promising drugs currently in development could emerge as new alternative therapies in the near future for patients with mCRPC, as we discuss below (Table 2).

### 4.1. DNA Repair Gene Mutations

Somatic mutations in DNA repair pathway genes occur in up to 23% of mCRPC tumors (19% of localized prostate tumors), with most mutations found in BRCA2 and ATM [25]. These mutations are often associated with germline mutations. One study found that 42% of patients with mCRPC (and 60% of patients with localized PCa) who had the BRCA2 mutation carried this mutation in their germlines [26].

Germline DNA repair mutations have been reported in approximately 10% of men with metastatic PCa [27]. In men with germline BRCA mutations, prostate cancer appears to develop earlier, with a more aggressive phenotype associated with significantly reduced survival times than in non-carriers [28]. Most scientific societies and clinical guidelines recommended genetic testing based on family history, histology, and risk groups for patients with PCa and any of the following (based on germline genetic testing): a positive family history, high-risk or very high-risk disease, regional or metastatic PCa (regardless of family history), Ashkenazi Jewish ancestry, and intraductal/cribriform histology. Germline testing should include MLH1, MSH2, MSH6, and PMS2 (for Lynch syndrome), and the homologous recombination genes BRCA2, BRCA1, ATM, PALB2, and CHEK2 [29,30]. Somatic tumor testing based on risk groups is recommended for patients with regional (N1) and metastatic PCa. Somatic testing should include BRCA1, BRCA2, ATM, PALB2, FANCA, RAD51D, CHEK2, and CDK12. In patients with metastatic castration-naïve and mCRPC, microsatellite instability (MSI-H) and mismatch repair-deficient (dMMR) should be included [29,30,31]. Genetic counseling and support are essential; when possible, pre-test counseling is advised, particularly in patients with a family history of the disease [31].

#### 4.1.1. PARP Inhibitors

PARP inhibitors, which have been approved for the treatment of other solid cancers such as ovarian cancer, are in advanced stages of development as a treatment for PCa. The mechanism of action of these agents is based on alterations in DNA repair mechanisms. As a treatment for mCRPC, the most advanced trial of PARP inhibitors is the phase III PROFOUND trial [32] involving patients with mCRPC with genetic alterations in DNA damage repair mechanisms (mainly BRCA1, BRCA2, and ATM). The PROFOUND trial compared olaparib (a PARP inhibitor) to the standard treatment selected by the study investigators. Patients in the experimental arm had a significantly better response rate (33% vs. 2%; OR: 20.86; 95% CI: 4.18 to 379.18, *p* < 0.001), PFS (7.4 vs. 3.6 months; HR 0.34; 95% CI: 0.25 to 0.47; *p* < 0.001), and OS (18.5 vs. 15.1 months). Subsequently, olaparib was approved by the United States Food and Drug Administration (FDA). Olaparib is now included in both the NCCN and APCCC guidelines [33,34], and germline and somatic testing for relevant alterations are now considered standard of care for these patients.

Recently, talazoparib was assessed in an open-label phase 2 trial (TALAPRO-1) in patients with mCRPC and DDR-HRR alterations [35]. In that trial, 128 patients (all of whom had received one or two taxane-based chemotherapy cycles and progressed on enzalutamide or abiraterone, or both) received oral talazoparib until disease progression. After a median follow-up of 16.4 months (interquartile range: 11.1–22.1), the objective response rate was 29.8% (95% CI: 21.2–39.6). The most common grade 3–4 treatment-emergent adverse events were anemia (31%), thrombocytopenia (9%), and neutropenia (8%). Serious adverse events were reported in 34% of patients. Talazoparib showed durable antitumor activity in these heavily pretreated patients with mCRPC and DDR-HHR gene alterations.

Several clinical trials are currently underway to assess the role of other drugs in the same class as olaparib (rucaparib and niraparib) for the treatment of mCRPC as well as in earlier stages of PCa. However, several questions remain to be resolved, including whether all genetic alterations in DNA repair mechanisms are predictors of the efficacy of PARP inhibitors and the clinical setting in which these drugs are likely to be most beneficial.

#### 4.1.2. Platinum-Based Chemotherapy for DNA Repair Defects

Emerging evidence suggests that the status of the DNA damage-repair pathway influences sensitivity to platinum-based chemotherapy. Patients with BRCA1 or BRCA2 mutations have a defect in homologous DNA recombination, making cells that harbor these mutations susceptible to DNA damaging agents, such as cisplatin or carboplatin [36]. A retrospective analysis of 141 men with mCRPC treated with ≥two doses of carboplatin and docetaxel at the Dana Farber Cancer Institute between 2001–2015 found that treatment benefits for patients with germline BRCA2 mutations [37]. Six of the eight BRCA2 carriers (75%) had a decrease in PSA > 50% within the first 12 weeks of starting this regimen compared to 23 of 133 non-carriers (17%) (*p* = 0.001). A decrease in PSA > 50% was associated with longer survival (18.9 months in BRCA2 carriers vs. 9.5 months in non-carriers). Several studies are currently underway to evaluate the role of platinum-based chemotherapy in patients with DNA repair defects.

### 4.2. Immunotherapy

Many investigators believe that PCa is immunologically cold (i.e., low immune sensitivity), as this type of cancer has fewer somatic mutations than other cancers and thus a lower immunogenicity level. Notwithstanding this unique characteristic, other features of PCa suggest that immunotherapy could be beneficial, including the slow growth of these tumors and the presence of antigenic expression of prostatic acid phosphatase (PAP), PSA, and prostate-specific membrane antigen (PSMA).

Although immunotherapy now forms part of the therapeutic arsenal for several cancers, its role in the treatment of PCa is not clear. The results of a phase III trial (IMPACT) comparing Sipuleucel-T—an autologous, dendritic cell-based vaccine—to placebo were published in 2010 [38]. Sipuleucel-T increased OS versus placebo (25.8 vs. 21.7 months; HR, 0.78; 95% CI: 0.61 to 0.98, *p* = 0.03) in asymptomatic or mildly symptomatic patients with mCRPC without prior chemotherapy. However, due to doubts about its efficacy, manufacturing challenges, and associated costs, this treatment has not yet been approved by any European regulatory agency. Although it has been approved in the USA by the FDA, its use in clinical settings remains relatively limited.

The use of targeted therapies such as cytotoxic T-lymphocyte antigen (anti-CTLA-4) or programmed cell death 1 (anti-PD-1) and its ligand (PD-L1), have not had much success to date in mCRPC. Phase III trials comparing ipilimumab [39,40] or anti-CTLA-4 to placebo in docetaxel-naive patients (28.7 vs. 29.7 months; HR, 1.11; 95.87%CI, 0.88 to 1.39; *p* = 0.3667) and in those who switched to docetaxel (11.2 vs. 10 months; HR, 0.85, 0.72–1-00; *p* = 0.053) show no improvement in survival. The results of the IMbassador250 trial [41], which compared enzalutamide alone to enzalutamide + atezolizumab in patients with mCRPC, were also negative (OS: 16.6 vs. 15.2 months; HR, 1.12; 95% CI: 0.91 to 1.37; *p* = 0.28).

The prevalence of high MSI-H/dMMR prostate cancer [40], and the clinical utility of immune checkpoint blockade are unknown. Pembrolizumab, an anti- PD-1 antibody, seems to be effective in MSI-H/dMMR CRPC. In one study, 54.5% of patients (6/11) treated with anti-PD-1/PD-L1 had a greater than 50% decline in PSA levels, and four of these patients also had a confirmed radiographic response [42].

Cyclin-dependent kinase (CDK12) alterations are reported to occur in 4–11% of PCa [25] cases and are more common in the mCRPC setting. Importantly, the CDK12-mutant genomic signature is mutually exclusive and distinct from dMMR subtypes of PCa. It has been hypothesized that focal tandem duplication-induced genomic instability represents a therapeutic vulnerability due to the formation of fusion-related immunogenic neoantigens [25]. If this hypothesis is validated, this would have implications beyond PCa, as it would represent a potentially novel tumor-agnostic application for immunotherapy [25]. However, although a large number of trials are currently evaluating these drugs, the findings reported to date do not show a significant impact on OS, which is why these agents are not included among the standard treatment options.

### 4.3. Radioisotopes

In recent years, interest in the role of antibodies—mainly those targeting PSMA linked to radioisotopes such as lutetium and actinium—has increased substantially. Lutetium-177 [¹⁷⁷Lu]Lu-PSMA-617 is a radiolabeled small molecule that delivers β radiation to cells expressing PSMA. This agent has been shown to present clinical activity in patients with mCRPC, and can be administered safely [43,44]. A phase II trial [45] comparing [¹⁷⁷Lu]Lu-PSMA-617 to cabazitaxel in patients with mCRPC showed that [¹⁷⁷Lu]Lu-PSMA-617 achieved a greater PSA response (65% vs. 37%, 95% CI: 16–42; *p* < 0.0001) with fewer grade 3/4 adverse events (33% vs. 53%). A recently published randomized open-label, phase III trial [46] (VISION trial) evaluated treatment with 177Lu-PSMA-617 in patients with mCRPC previously treated with at least one androgen receptor pathway inhibitor and one or two taxane regimens. All patients had a PSMA-positive gallium-68 (68Ga)–labeled PSMA-11 positron emission tomography-computed tomography scan. The alternate primary end points were imaging-based PFS and OS. Of the 1179 screened patients, 831 underwent randomization. The median follow-up was 20.9 months. Compared to standard care alone, the addition of 177Lu-PSMA-617 to standard care significantly prolonged both imaging-based PFS (median: 8.7 vs. 3.4 months; HR for progression or death, 0.40; 99.2% CI, 0.29 to 0.57; *p* < 0.001) and OS (median: 15.3 vs. 11.3 months; HR for death, 0.62; 95% CI, 0.52 to 0.74; *p* < 0.001). The incidence of ≥grade 3 adverse events was higher with 177Lu-PSMA-617 than without it (52.7% vs. 38.0%), but QoL was not adversely affected.

Based on the results of that trial, the authors concluded that [¹⁷⁷Lu]Lu-PSMA-617 is an effective new therapeutic class and a potential alternative to taxanes regimens.

### 4.4. AKT Inhibitors

AKT is part of the PI3K-AKT-mTOR pathway, acting as a regulator of cellular metabolism. Loss of function of the PTEN tumor suppressor, resulting in dysregulated activation of the PI3K signaling network, is recognized as one of the most common driving events in prostate cancer development. PTEN loss is highest in aggressive metastatic disease, and therefore, efforts to find available inhibitors of specific components of the PI3K/PTEN/TOR signaling network in PCa treatment is a challenge [47].

The results of the phase III IPATential150 trial were recently presented at the annual congress of the European Society of Medical Oncology [48]. That trial compared first-line abiraterone + placebo to abiraterone + ipatasertib (an AKT inhibitor) in patients with mCRPC. Radiographic PFS in patients with PTEN loss (the primary outcome measure) was significantly better in the combined treatment arm (HR, 0.39; 90% CI, 0.22–0.7). Although follow-up is still ongoing, the available data in the intention-to-treat population shows no significant between-group differences in radiographic PFS or OS (18.9 vs. 15.6 months, HR, 0.72; 90% CI, 0.47–1.11, *p* = 0.22). However, these data suggest that, in the future, PTEN status may play an important role in selecting potential candidates for this therapeutic strategy.

## 5. Therapeutic Sequencing in mCRPC

At present, for most patients, no clear predictors of response are available, which makes it difficult to determine the most suitable treatment for each patient to consider personalized treatment. Exceptions to this include BRCA1-2 mutations and alterations in genes related to PARP inhibitors or PTEN loss, as well as MSI-H mutations.

Currently, there is no optimal therapeutic sequence for all patients. Numerous factors must be considered in each case, including the patient’s overall health, the presence of comorbidities, previous treatments (and the efficacy and toxicity thereof), patient preferences, and the availability of clinical trials at each center. As a result, although general recommendations can be made, these must be tailored to the needs and characteristics of the individual patient. In fact, the clinical evidence shows that the patient’s individual characteristics (e.g., performance status; comorbidities; degree of frailty; and symptoms) play a crucial role in treatment selection. Thus, the various treatment options, including the efficacy and toxicity profile of the drug, should be evaluated only after carefully considering the patient’s individual clinical profile.

In the clinical trials conducted to date, first-line treatment for patients with asymptomatic or mildly symptomatic mCRPC has generally involved novel hormonal agent such as abiraterone or enzalutamide (when not contraindicated). However, in certain cases—such as in patients with high Gleason scores, visceral involvement, a short-lived response to prior androgen deprivation therapy, or only slightly elevated PSA levels with large tumor volumes—direct treatment with chemotherapy (docetaxel) can be considered given that those factors are usually associated with a poor response to hormonal therapy [49].

The available data suggest that the sequential delivery of two different hormonal agents provides little benefit. Numerous phase II trials have shown that sequential administration of abiraterone and enzalutamide (regardless of the order and regardless of previous treatment with another chemotherapy agent) is associated with poor PSA response and minimal gains in PFS [50]. These findings were confirmed in 2019 with the publication of the phase III CARD trial [51]. That trial involved patients with mCRPC who had been previously treated with docetaxel and either abiraterone or enzalutamide who developed disease progression within 12 months of receiving these agents. The patients were randomized to receive cabazitaxel (25 mg/m2 intravenously every 3 weeks) plus daily prednisone and granulocyte colony-stimulating factor or the other inhibitor (either 1000 mg of abiraterone plus prednisone daily or 160 mg of enzalutamide daily). The cabazitaxel arm had better OS (13.6 vs. 11 months; HR, 0.64; 95% CI: 0.46 to 0.89; *p* = 0.008), PFS (4.4 vs. 2.7 months; HR, 0.52; 95% CI: 0.40 to 0.68; *p* < 0.001), and PSA response rates (35.7% vs. 13.5%) than placebo. Pain control was also better with cabazitaxel. The ≥G3 adverse event rate was similar (56.3% vs. 52.4%). The findings of that trial confirmed that these patients obtain a greater benefit from chemotherapy than from a second hormone therapy. Despite those findings, we do not recommend hormonal therapy sequencing in most mCRPC patients. At the end, the effects of therapeutic sequencing in PCa can be affected by incorporating several of these agents during earlier stages of the disease (metastatic hormone-naive PCa or non-metastatic CRPC) [52].

## 6. Bone-Targeted Therapy

Prostate cancer tropism towards the bone explains high incidence rate (90%) of bone metastases in the castration-resistant phase. Preventing skeletal-related events (SRE) is crucial to avoid pathologic fractures, medullar compression, and the need for additional treatment (radiotherapy and/or surgery), all of which are associated with increased morbidity and lower QoL in these patients.

Zoledronic acid, a potent third-generation IV bisphosphonate (inhibitor of osteoclastic activity) has been shown to significantly reduce the risk of SREs in patients with mCRPC (44% vs. 32% with placebo) [53] and may even delay the emergence of these events. Although this drug was approved by regulatory agencies in 2004, it has not been shown to improve survival outcomes [53].

Denosumab is a human monoclonal antibody to RANKL (an osteoclastosis activator) that significantly suppresses bone resorption. It has been approved by the FDA and the EMA for the prevention of SREs in patients with bone metastases from solid tumors. However, similar to zoledronic acid, it does not improve OS. A study comparing zoledronic acid to denosumab found that the latter drug was superior in terms of toxicity profile [54].

## 7. Conclusions

The treatment of metastatic castration-resistant prostate cancer has changed considerably in recent years with the development of systemic treatments that improve both survival and QoL outcomes. However, due to a lack of validated biomarkers and robust predictors to help select the optimal treatment, these systemic therapies must be selected on a case-by-case basis in accordance with the patient’s individual characteristics and preferences. Treatment selection must also consider prior therapies and access to clinical trials. The therapeutic arsenal for mCRPC is expected to change significantly in coming years as several new treatments and novel biomarkers are incorporated into routine clinical practice. Ultimately, these developments are expected to allow clinicians to offer patients a truly personalized treatment approach.

## Figures and Tables

**Table 1:** Current treatments in mCRPC.

Therapy	Type	Study Design	Trial Name	N Patients	Trial Phase	Efficacy OS (Months)	Adverse Events G3-4	Comments
Chemotherapy	Docetaxel	MTX + PRD vs. DOC + PRD	TAX327	1006	III	18.9 vs. 16.5	26%	DOC improves OS and symptoms
	Cabazitaxel	CBZ + PRD vs. MTX + PRD CBZ20 + PRD vs. CBZ25 + PRED vs. DOC	TROPIC FIRSTANA	755 1168	III III	15.1 vs. 12.7 25.2 vs. 24.3	82% neutropenia 41.2–60.1%	CBZ improves OS after DOC-based therapy CBZ20/25 is not superior to DOC
Second-generation antiandrogens	Abiraterone	AB + PRD vs. Placebo + PRD AB + PRD vs. Placebo + PRD	COU/301 COU/302	1195 1088	III III	14.8 vs. 10.9 34.7 vs. 30.3	55% vs. 43% 48% vs. 42%	AB improves OS after DOC treatment AB improves OS in chemotherapy-naive patients
	Enzalutamide	ENZA vs. Placebo ENZA vs. Placebo	AFFIRM PREVAIL	1199 1717	III III	18.4 vs. 13.6 32.4 vs. 30.2	45% vs. 53% 43% vs. 37%	ENZ improves OS after chemotherapy ENZ improves OS in chemotherapy-naïve patients
Radiopharmaceuticals	Radium-223	Ra-223 vs. Placebo Ra-223 + AB vs. AB	ALSYMPCA ERA	921 806	III III	14.9 vs. 11.3 30.7 vs. 33.3	56% vs. 62% 28.6% vs. 11.4% *	Ra-223 improves OS Ra-223 + AB did not improve SSE-free survival and increased bone fractures

Key: N, number; AB, Abiraterone; CBZ, Cabazitaxel; ENZA, Enzalutamide; DOC, Docetaxel; MTX, Mitoxantrone; PRD, Prednisone; OS, Overall survival; SSE, Symptomatic skeletal event. * Bone fracture risk.

**Table 2:** Emerging therapies in mCRPC.

Therapy	Type	Study Design	Trial Name	N Patients	Trial Phase	Efficacy OS (Months)	Adverse Events G3-4	Comments
PARP inhibitors	Olaparib	Olaparib vs. ENZA or AB	PROFOUND	387	III	18.5 vs. 15.1	51% vs. 38%	Olaparib improves PFS, measures of response and patient-reported end points in those with alterations in genes with a role in homologous recombination repair
Immunotherapy	Sipileucel-T	Sipileucel vs. placebo	IMPACT	512	III	25.8 vs. 21.7	31.7% vs. 35.1%	Sipuleucel-T improves OS. No effect on time to disease progression
	Ipilimumab	RT + Ipilimumab vs. RT + Placebo Ipilimumab vs. Placebo	CA184-043 CHEMO-NAIVE	799 602	III III	11.2 vs. 10.0 28.7 vs. 29.7	26% vs. 3% 40% vs. 6%	NO differences in OS Ipilimumab did not improve OS, but did improve PFS and PSA response
	Atezolizumab	Atezolizumab + ENZA vs. ENZA	IMBASSADOR 250	759	III	16.6 vs. 15.2	54% vs. 35%	Atezolizumab did not improve OS
Radioisotopes	[¹⁷⁷Lu]Lu-PSMA-617	[¹⁷⁷Lu]Lu-PSMA-617 vs. CBZ [¹⁷⁷Lu]Lu-PSMA-617 vs. Standard care	THERAP VISION	291 1179	II III	65% vs. 37% * 15.3 vs. 11.3 8.7 vs. 3.4 ***	33% vs. 53% 52.7% vs. 38%	[¹⁷⁷Lu]Lu-PSMA-617 had better PSA response and fewer toxicity than CBZ [¹⁷⁷Lu]Lu-PSMA-617 prolonged PFS and OS
AKT inhibitors	Ipatasertib	Ipatasertib + Placebo vs. Ipatasertib + AB	IPATENTIAL 150	253	II	18.9 vs. 15.6	64.3%, 50.6% ** vs. 35.4%	Ipatasertib + AB showed better antitumor activity in PTEN-loss tumors

Key: * PSA response rate; ** Ipatasertib, 400 mg and 200 mg, respectively; *** PFS; AB Abiraterone; CBZ Cabazitaxel; ENZA Enzalutamide; PFS Progression-free survival; PSA Prostate-specific antigen.

## Data Availability

Data sharing not applicable. No new data were created or analyzed in this study.
